# What makes interventions aimed at improving dietary behaviours successful in the secondary school environment? A systematic review of systematic reviews

**DOI:** 10.1017/S1368980022000829

**Published:** 2022-09

**Authors:** TE Capper, SF Brennan, JV Woodside, MC McKinley

**Affiliations:** Centre for Public Health, Queen’s University Belfast, Institute of Clinical Sciences, Grosvenor Road, Belfast BT12 6BJ, UK

**Keywords:** Adolescence, Diet, Secondary schools, Food choice, Systematic review

## Abstract

**Objective:**

To systematically review evidence from systematic reviews of interventions to improve dietary behaviours and reduce food wastage in secondary school pupils.

**Design:**

CINAHL, Cochrane Reviews, EMBASE, MEDLINE, PsychINFO and Web of Science were searched for systematic reviews of school-based dietary interventions from 2000 to 2020 published in a peer-reviewed journal in English. Articles were reviewed independently by two authors. AMSTAR-2 was used for quality assessment.

**Setting:**

Secondary school dietary interventions.

**Participants:**

Adolescents (aged 11–18).

**Results:**

In total, thirteen systematic reviews of dietary interventions in secondary schools met the inclusion criteria. A number of key characteristics of interventions that contributed to improvements in food choices in secondary school pupils were identified. These included the combination of education and environmental restructuring, incorporation of computer-based feedback, media or messaging, peer and/or parent involvement, an increase in the availability of healthy foods and the use of behavioural theory as a basis to the intervention. Intervention components that contributed specifically to a reduction in sugar-sweetened beverage intake or an increase in fruit and vegetable consumption, which are particularly relevant to adolescents, could not be determined. Similarly, evidence for interventions that improve nutritional knowledge and attitudes was limited.

**Conclusions:**

This systematic review of systematic reviews has identified a number of components of dietary interventions that can be explored to improve dietary behaviours in secondary school environments and, if demonstrated to be effective, be considered for inclusion in policies and strategies to improve the school food environment and promote dietary change.

The transition from childhood to adolescence is often associated with less healthy dietary choices^(1,2)^, commonly a reduction in fruit and vegetables (FV) intake, and an increase in consumption of sugar-sweetened beverages (SSB)^(1,3)^. Data suggest that these and other unhealthful dietary behaviours track into adulthood and may lead to an increased risk of obesity and related disease in later life^(4)^. It is, therefore, critical that both children and adolescents have the knowledge and ability to make positive food choices and develop good dietary habits that can be carried into adulthood.

Through food provision, nutrition education and healthy school policies, schools can create an environment promoting and enabling healthful dietary choices, with nutrition education embedded into a variety of subjects including science and health^(5)^. However, school food provision can also be associated with large amounts of food waste^(6–9)^, which has a negative impact not only on the environment^(10)^ but also on the nutritional benefit of the food provided to the pupils. Low food waste production in schools may be an indicator of a well-functioning system and positive food choices in the pupils. The school setting also provides a unique research opportunity to engage with children and adolescents across diverse socio-economic and ethnic backgrounds. It is not surprising, therefore, that there are large numbers of dietary intervention studies carried out in schools.

In line with the abundance of nutrition studies in schools, the publication of systematic reviews (SR) has risen in an attempt to summarise the evidence gathered from these interventions. Fundamental in translating the evidence into practical solutions that improve the diet is identification of the intervention components and characteristics associated with effectiveness. However, published SR differ in their scope and intervention focus, complicating the identification of characteristics that lead to improved dietary behaviours. Furthermore, primary and secondary school pupils differ in the freedom of food choice afforded to them, access to ‘competitive foods’ from vending machines or offsite outlets^(11)^ and the cognitive development processes and social interactions associated with their age^(12)^, but are often grouped in reviews. The variation in the primary and secondary school systems means that effective components of dietary intervention studies may differ between educational levels. Therefore, studies in primary and secondary schools should be considered independently in order to reveal successful interventions for the appropriate age group. To date, a number of reviews of SR that have been published on obesity prevention or healthy eating interventions combine schools with other settings and focus on a wide age range^(13,14)^, making it difficult to elucidate successful components of school-based interventions for adolescents. A WHO report on food and nutrition policy for schools was published in 2006 and although it provides separate dietary recommendations and suggested food preparation skills for younger and older pupils, it does not consider which intervention components are most relevant to each age group^(15)^.

This paper, therefore, systematically reviews published SR to summarise the evidence base on dietary interventions and food wastage in the secondary school environment. It adds to the literature by synthesising key findings from these reviews to consolidate successful components upon which secondary school food interventions can be based. This paper aims to identify intervention components targeting dietary behaviours specifically relevant to adolescents (aged 11–18). Ultimately, this could inform the development and implementation of policies and strategies aimed at improving food choices in secondary school pupils.

## Methods

### Inclusion/exclusion criteria

To be included in this review, SR had to meet the following criteria: (i) published in a peer-reviewed journal before May 2020; (ii) published in the English language; (iii) review school-based interventions; (iv) involve secondary school pupils (adolescents between the ages of 11 and 18) and (v) describe the effect of intervention or school policy on food choice, dietary behaviours or food waste. In addition, SR were excluded that: were conducted in clinical adolescent populations, i.e. overweight or obese; did not report results of school interventions independently if multiple settings were described; did not report results from adolescents aged 11–18 independently if interventions in younger age groups were also included; did not report dietary behaviours independently if other health behaviours were studied and were narrative reviews, reports or position statements. If studies on primary/elementary school pupils were included in the SR, they were included if there was a subgroup analysis for the secondary school pupils. Where possible, age limits were applied to database searches to reflect adolescents. SR were included if they were published after 2000 to ensure that included reviews reflected current or recent school policies and practices and the contemporary school environment.

### Study selection

CINAHL, the Cochrane Database of Systematic Reviews, EMBASE, MEDLINE, PsychINFO and Web of Science were searched (see Additional File 1 for search strategy). An initial database search in May 2018 was updated in May 2020. Two authors independently reviewed all titles generated by the search and removed duplicates. These articles were then subjected to abstract review independently by two authors, and full texts of potentially relevant articles were obtained. Discrepancies regarding relevance of the full texts for inclusion were resolved by discussion with a third author. Reference lists of the remaining articles were searched to retrieve any additional relevant articles.

### Data extraction

Data were extracted against a template by one author and checked by the other authors. Relevant data were extracted from identified reviews using the following elements: aim, inclusion criteria, search period, geographical region of included studies, number and type of study, intervention approaches used in the included studies, main results of the SR, as well as any results specifically related to intervention approach, e.g. environmental restructuring *v*. education only, peer or parental involvement, intervention intensity, intervention provider and theoretical basis of includes studies. All results were reported as extracted from the original research paper; authors did not refer back to the primary studies.

### Quality assessment

To determine the quality of the included SR, the AMSTAR 2 (A Measurement Tool to Assess Systematic Reviews) was applied^(16)^ (Additional File 2). Through discussion amongst the authors prior to quality assessment, three critical domains of the AMSTAR 2 were agreed. These were (1) explanation of selection of the study designs; (2) use of a comprehensive literature search strategy and (3) account for risk of bias in individual studies when interpreting/discussing the results. These three domains were thought to be most relevant to quality assessment of studies in this field. The included SR were then assessed based on adherence to these critical domains, as well as the presence of non-critical weaknesses determined by their relevance to the current topic and SR included. SR were marked ‘low’ or ‘critically low’ if they failed to address one or more than one of the critical domains respectively, as guided by AMSTAR 2. Two questions (7 and 10) were deemed irrelevant to the topic through initial discussions amongst the authors and, therefore, SR were not penalised if they failed to address these questions. Subsequently, SR with no or one relevant, non-critical flaw were deemed to be of ‘high’ quality and SR with more than one relevant, non-critical flaw were deemed to be of ‘moderate’ quality. Two authors independently conducted the quality assessment and any disagreements were discussed with the other authors until consensus was reached.

### Data synthesis

Statistical analyses or meta-analyses were not conducted due to the heterogeneity in outcomes among SR. Instead, the authors extracted the results of existing analyses in the SR and reported them in a systematic format. In accordance with reporting of SR, PRISMA (Preferred Reporting Items for Systematic Reviews and Meta-Analyses) guidelines were followed^(17)^ (Additional File 3).

## Results

**Fig. 1:**
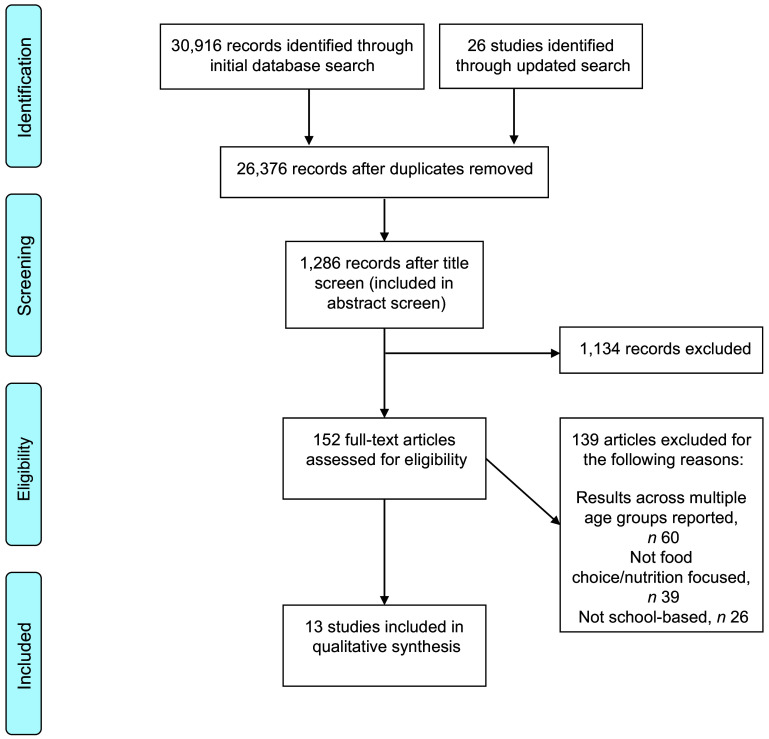
PRISMA flow chart of the systematic review process

The study selection process is outlined in Fig. 1. Thirteen SR on food choice and dietary behaviours met the inclusion criteria. No SR reporting outcomes related to food wastage in the secondary school environment were found.

### Systematic review characteristics

Table 1 presents the main characteristics of the SR. They varied considerably in their focus and inclusion criteria. Three SR examined the effect of general healthy eating promotion in schools on dietary behaviours^(18–20)^, three examined the impact of nutrition education interventions^(21–23)^, three focused on FV interventions^(24–26)^, two focused on beverage intake (SSB or water)^(27,28)^, one examined interventions using the WHO Health Promoting Schools framework^(29)^ and one explored barriers and facilitators for healthy eating^(30)^.

**Table 1:** Characteristics of included systematic reviews

Reference	Time period of search	Geographical region of studies	Aim as described by the SR	Inclusion criteria	Key diet-related outcome(s) of SR
*High quality*					
Shepherd *et al.* (2006)^(30)^	1990–2001	States UK only, however in 7 outcome evaluations described – 4 in USA, 3 in Europe (1 each in UK, Norway and Finland)	Undertake a ‘systematic mapping’ of research on the barriers and facilitators for healthy eating among young people, especially those from socially excluded groups	*Design*: UK only, healthy eating as a main focus with a comparison or control group	Changes in nutrition habits, dietary composition (salt and saturated fat), FV intake, or nutritional knowledge
				*Participants*: aged 11–16	
McHugh *et al.*, (2020)^(29)^	2013 to most recent	6 studies in the USA, 1 each in Ecuador, Belgium, Finland, France, Australia and India	To examine the effectiveness of interventions using the WHO HPS framework approach in increasing PA and improving diet of 11–18 year olds	*Design:* RCT clustered at the level of school, district or geographical area and interventions aimed at changing diet and/or physical activity, which addressed all the components of the WHO HPS framework	Change in self-reported or objectively measured diet outcomes (e.g. FV consumption)
				*Participants:* aged 11–18	
*Moderate quality*					
Van Cauwenberghe *et al.* (2010)^(18)^	Jan 1990–Dec 2007	Europe	To compile evidence on the effectiveness of school-based programmes promoting healthy diets on dietary intake and anthropometric measurements in children and adolescents in Europe	*Design*: main or one component was promotion of a healthy diet (no restriction on study design)	Changes in dietary behaviours
				*Participants*: aged 6–18 in the EU	
*Low quality*					
Knai *et al.* (2006)^(24)^	No limits applied	USA	To collect and summarize worldwide evidence from published and “grey” literature on current evaluations of all interventions and programmes which promote fruit and vegetable consumption in children	*Design:* all interventions and promotion programmes where the primary outcome (FV intake) was measured with presence of a control group	Change in FV intake
				*Participants*: aged 5–18	
De Sa and Lock. (2008)^(25)^	Earliest record to 2007	3 Europe, 4 USA	Systematically synthesise world-wide evidence from published and unpublished literature on interventions to promote fruit and/or vegetable consumption in children in school settings	*Design:* presence of a control group and follow-up period of at least 3 months	Change in intake of FV, or a change in knowledge, attitude or preference to FV
				*Participants*: <18 years	
Meiklejohn *et al.*, (2016)^(22)^	2000–2014	2 in the USA; 1 each in Belgium, Greece, Finland, Norway, Australia and Sweden; 1 across Norway, Spain and The Netherlands	To update evidence on the impact of multi-strategy nutrition education interventions on adolescent’s health and nutrition outcomes and behaviours.	*Design:* multi-strategy RCT including nutrition education	Changes in biochemical markers and dietary composition i.e. FV, fat, sugar, SSB
				*Participants*: aged 10–18	
Nørnberg *et al.* (2016)^(26)^	No limits applied	7 in the USA, 1 in Canada, 1 in Denmark	To identify and assess the quality of studies investigating attitudes towards choice architectural nudge interventions and the effects of interventions on promoting vegetable consumption among school-attending adolescents	*Design:* intervention or experimental design applying choice architectural nudging	Changes in dietary intake, food choice or attitude towards vegetables
				*Participants:* aged 11–19	
Vézina-Im *et al.*, (2017)^(27)^	Before Dec 2016	24 in USA, 3 in Canada, 2 in Australia, 3 in Europe, 1 each in Brazil, China, India and Korea	Perform a SR of school-based interventions aimed at reducing SSB consumption among adolescents (1) and identify the BCTs most effective at decreasing SSB consumption in order to inform future school-based interventions aimed at changing this behaviour among adolescents (2)	*Design*: RCT, quasi-experimental, one-group pre-post	Changes to SSB consumption
				*Participants*: aged 12–17	
Murimi *et al.* (2018)^(21)^	2009–2016	USA and non-USA	Identify the characteristics associated with successful nutrition education interventions in children	*Design*: RCT, pre-post or quasi-experimental	Change in biochemical measurements, dietary intake, knowledge, preference, attitude, behaviour, dietary diversity score, or food and beverage availability at school
				*Participants*: aged 2–19	
Calvert *et al.* (2019)^(19)^	1987–2016	10 in the USA, 10 in Europe, 3 in Australia, 2 in Canada, 1 each in China, Israel, Taiwan and Tunisia	Evaluate the effectiveness of school-based interventions in improving dietary behaviour (1) and identify intervention characteristics that may contribute to the effectiveness of school-based dietary behaviour change interventions (2)	*Design*: at least one pre- and post-intervention comparison of dietary behaviour	Changes in dietary behaviours including increasing FV consumption, snacking, SSB intake or daily fat and sugar intake
				*Participants*: aged 11–16	
*Critically low quality*					
Ajie *et al.* (2014)^(23)^	Jan 2002–Aug 2013	Not reported but some European and USA studies mentioned	To evaluate the overall effectiveness of computer-based interventions that provided nutrition education related to adolescent overweight prevention or treatment	*Design:* RCT, quasi-experimental or intervention with no concurrent control	Changes in diet composition or nutritional knowledge
				*Participants*: aged 12–18	
Robinson *et al.* (2014)^(20)^	Jan 1980–Mar 2013	USA	Assess the evidence for school-based interventions that promote healthy eating and physical activity in African American children and adolescents	*Design*: RCT, controlled trials, quasi-experimental	Changes in dietary composition, nutritional knowledge, nutrient intake, FV preferences, or food habits
				*Participants*: aged 3–15, African Americans	
Vézina-Im *et al.* (2019)^(28)^	No limits applied but all in last 10 years	7 in the USA, 1 each in Australia, Greece, Belgium and the UK	To summarize the results of the latest scientific literature on determinants of water consumption and interventions to promote water consumption among adolescents	*Design:* interventions to promote water consumption	Changes in water consumption, SSB/milk/fruit juice/hot beverage/alcoholic drink intake, or FV intake
				*Participants:* aged 12–17	

FV, fruit and vegetable; HPS, health-promoting schools; RCT, randomised-controlled trial; SR, systematic review; SSB, sugar-sweetened beverages.

Two SR included only randomised-controlled trials^(22,29)^, while one stipulated no restrictions on intervention design^(18)^, resulting in a wide range in the number of included studies (Table 2). In general, the reviews did not evaluate which primary studies held greater weight based on study design and methodological rigour. Results from 168 primary studies in secondary schools were included in the SR. There was little overlap in the primary studies included Haerens *et al.* (2006)^(31)^ appeared in five SR^(19,22,23,29)^, and Lytle *et al.* ^(32)^ and Haerens *et al.* (2007)^(33)^ were both included in four SR^(22,24,25,29)^ and^(18,22,23,25)^. All SR included interventions carried out in both males and females. Eight SR examined interventions in secondary schools only, while five also included interventions in pre- and primary schools but reported results for each educational level independently, thus allowing inclusion in this SR. Most SR that included a range of age groups from pre-school to secondary school^(18,20,21,24,25)^ had a higher number of studies in primary schools compared with secondary schools. Eighty-nine percentage of primary studies were conducted in North America and Europe, with Australia, the Middle East, South and East Asia and South America represented in a small number of studies (Table 1).

**Table 2:** Intervention effectiveness as reported in the systematic reviews

Study	N of studies (N participants)	Duration of intervention and/or follow-up time	Behaviour change approach	Effectiveness	Results related to behaviour change approach
*High quality*					
Shepherd *et al.* (2006)^(30)^	**7**	Intervention period not consistently reported; Immediate to 5 y follow-up	Information provision, environmental restructuring, trained parents about nutrition, health-screening resources, personalised feedback, social support	Six out of seven were effective at improved healthy eating behaviour and/or knowledge. The evidence from the well-designed evaluations of the effectiveness of the healthy eating initiatives is mixed	Increasing availability of healthy food (two studies) was effective at improving healthy eating; peer-led intervention (three studies) was effective. Half of sound interventions involved parents in the education but difficulty in securing parent attendance
	5 RCT (268 to 4253)				
	2 non-RCT (sixteen and one not described)				
McHugh *et al.* (2020)^(29)^	**12** RCT (462 to 25 000)	5 w to 3 y intervention period; 1 y to 3 y follow-up	Information provision, environmental restructuring, social support	One out of four nutrition-only interventions reported a significant change in overweight. Three out of five nutrition and PA interventions reported significant changes in BMI or obesity; no changes in dietary intakes. Threee out of three PA-only interventions reported significant changes in BMI, fitness and MVPA	Each of the HPS interventions used a combination of BCT, so effectiveness of each could not be determined. Increasing the availability of healthy food had no effect but restriction of unhealthy food reduced sucrose intake
*Moderate quality*					
Van Cauwenberghe *et al.* (2010)^(18)^	**13 (in adolescents)**	One-off to 2 y intervention period; follow-up 2 w to 2 y	Environmental structuring, information provision, school nutrition policy, peer leaders, parental involvement, school staff involvement	Ten out of thirteen studies reported overall improvements in dietary behaviours, one reported no effect and two reported mixed results	Moderate evidence found for the effect of education-only. Inconclusive for the effect of environmental restructuring. Limited evidence for multicomponent programmes on dietary behaviour
	5 RCT (54 to 1613)				
	5 non-RCT (228 to 2965)				
	1 B-A (475)				
	2 PC (158 to 21 305)				
*Low quality*					
Knai *et al.*, (2006)^(24)^	**4 (in adolescents)**	Intervention period not reported; 2–3 year follow-up	Integrated curriculum, goal setting, environmental restructuring, teacher training, peer leaders, parental involvement, school food service staff involvement, nutrition policy, community involvement	One out of four studies reported positive results (+0·32 servings/day of FV) in girls	Social support (parents and peers) was deemed important to the success of interventions, as was an increased exposure to FV. However, it is not clear if this applies to both primary and secondary schools equally
	4 RCT (>1000)				
De Sa *et al.*, (2008)^(25)^	**7 (in adolescents)** 6 RCT (12 267) 1 non-RCT (99)	12 w to 3 y intervention period; follow-up 3 to 24 mo	Environmental restructuring, information provision, school policy, teacher training, peer leaders, parental involvement, school food service staff involvement, community involvement	Five out of seven studies reported significant increases in fruit and/or vegetable consumption. One study reported increases in intake during the intervention, which was not sustained at follow-up. One found decrease only in fat intake	Environmental change only (FV provision) – 1 positive result and 1 no change. Environmental plus education – higher FV intake following interventions but not always maintained at follow-up and 1 only increased in girls
Meiklejohn *et al.* (2016)^(22)^	**11** 11 RCT (191 to 3503)	12 w to 4 school terms intervention period; follow-up not consistently reported	Information provision, environmental restructuring, parental involvement, peer leaders, goal setting, prompts to reinforce behaviour, tailored computer-based feedback	Nine out of eleven studies reported significant changes in dietary intake. Components of the interventions that showed statistically significant changes in dietary intake included facilitation of the programmes by school staff and teachers, parental involvement and using theoretical models to guide the intervention’s development	Environmental changes such as to the canteen and vending machines were associated with significant changes in dietary intake. Educational interventions were successful when they were behaviourally-focused, inclusive of theory, involved parents and were delivered by school staff and teachers
Nørnberg *et al.* (2016)^(26)^	**9**	1 d to 1 year intervention period; follow-up not reported	‘Nudging’ involving environmental restructuring, free provisioning, serving style	Two out of nine studies reported significant increases in vegetable intake. Two further studies reported positive changes but statistical significance was not reported	Free provisioning (four studies) did not significantly increase vegetable consumption but had an effect on attitudes and willingness to try. Serving style (five studies) had mixed effects
	1 RCT (1277)				
	4 PP (288 to 851)				
	1 QE (3690)				
	1 CO (156)				
	1 EXP (138)				
	1 EVAL (1127)				
Vezina-Im *et al.* (2017)^(27)^	**36**	Variable and not consistently reported	Providing information about health consequences, environmental restructuring, behavioural goal setting, self-monitoring, threat to health and social support	Twenty-six out of thirty-six studies were effective in decreasing SSB consumption. Nnie out of ten legislative/environmental studies reporting a significant reduction in SSB. Thirteen out of twenty educational/behavioural and four out of six combination were effective	Not possible to identify the most effective BCT, as studies often used a combination in their experimental group. The majority of environmental intervention used restructuring. Educational interventions most often provided information about health consequences or threat to health
	13 RCT (82 to 5219)				
	12 one-group PP (38 to 65 000)				
	11 QE (101 to 2292)				
Murimi *et al.* (2018)^(21)^	**8 (in adolescents)**	4 w to 1 school year intervention period; follow-up rarely reported	Environmental restructuring, information provision, peer leaders, counselling, parental involvement, school policy	Six out of eight studies reported significant improvements in dietary behaviours and knowledge. One study reported significant improvements in high-density lipoproteins and significant increases in nutritional knowledge in grades 7/8 but not in other grades. One study reported significant improvements in overweight/obesity and biochemical data suggestive of metabolic syndrome	Successful interventions added policy and restructured the environment, aligned activities with objectives, used age-appropriate activities, provided the intervention frequently, engaged with parents face-to-face and trained implementers to ensure fidelity
	2 RCT (510 to 3110				
	2 PP (181 to 263)				
	3 QE (100 to 4003)				
	1 LS (233)				
Calvert *et al.* (2019)^(19)^	**29**	2 w to 3 school year intervention period; follow-up immediately or 6 w to 4 y	Environmental restructuring, peer involvement, educational media, tailored computer-based feedback, nutritional handbooks, practical lessons	Twenty-four out of twenty-nine studies reported significant improvements in dietary behaviour	Increased availability of healthy food had a significant positive effect on dietary behaviours. Peer involvement, educational media, increased in-school availability of healthy foods and tailored computer-based feedback were associated with improvements in dietary behaviour
	19 RCT (98 to 4603)				
	7 QE (88 to 4003)				
	3 CO (344 to 32 482)				
*Critically low*					
Ajie *et al.* (2014)^(23)^	**10** 6 RCT (304 to 2287) 3 QE (103 to 275) 1 randomised no control (366)	Average 6·5 sessions; range from one-off to sixteen sessions	Information provision, self-monitoring, personalised feedback, social support, parental involvement, motivational messages, peer leaders, personalised feedback, environmental restructuring, dietary counselling	Six out of ten studies had significant positive effects on nutrition- or obesity-related variables, with small changes in diet, physical activity, knowledge and self-efficacy	Skill-building strategies and parental involvement affected the outcomes of the interventions
Robinson *et al.* (2014)^(20)^	**8 (in adolescents)** 3 RCT (221 to 2120) 5 QE (48 to 2132)	1 mo to 2 y intervention period; only one study states follow-up of 2 y	Information provision, teacher training, peer leaders, environmental restructuring	Five out of eight studies reported significant positive changes in knowledge and/or dietary behaviours	Studies that incorporated the lunchroom setting and school nutrition reported positive outcomes in altering dietary behaviours while those that did not include the environment demonstrated smaller but positive change


Vezina-Im *et al.* (2019)^(28)^	**11**	5 d to 2 y	Environmental restructuring, prompts/cues, health consequences, goal setting, problem solving, self-monitoring, instruction provision, behaviour substitution, social support, persuasive argument	Eight out of thirteen reported significant increases in water consumption (1 reported increase only in adolescents <17 years)	Educational only interventions reported no significant change in water consumption. 75 % of interventions with both educational and environmental components reported a significant increase in water consumption
	7 RCT (38 to 2997)				
	4 QE (92 to 2965)				

AA, African American; B-A, before–after; CO, cohort study; CT, crossover trial; EVAL, evaluation using post-test survey; EXP, experimental intervention; FV, fruit and vegetable; LS, longitudinal study; MVPA, moderate-vigorous physical activity; PA, physical activity; PC, prospective cohort; PP, pre-post; QE, quasi-experimental; RCT, randomised-controlled trial; SSB, sugar-sweetened beverage.

### Study quality

Two SR were rated ‘high’^(29,30)^; one was rated ‘moderate’^(18)^, seven were rated ‘low’^(19,21,22,24–27)^ and three were rated ‘critically low’^(20,23,28)^. The critical weaknesses identified in the low and critically low-quality SR were a lack of explanation for the study designs included in the SR and not accounting for risk of bias in interpretation/discussion of results. For moderate quality SR, the authors did not register or report a pre-designed protocol or perform data extraction in duplicate.

### Evidence synthesis

#### Reported effectiveness at improving dietary behaviours

Of the three SR that examined the effect of general healthy eating promotion, two low-quality SR^(19,20)^ concluded that there was evidence for improvement, and one moderate quality SR^(18)^ concluded that there was moderate and limited evidence for the effectiveness of educational and multicomponent interventions, respectively. For nutrition education interventions^(21–23)^, authors concluded that there was evidence for effectiveness based on positive results in the majority of primary studies included, provided that they involved certain intervention components; two SR were of low quality and one was of critically low quality. Of the SR that focused on FV intake^(24–26)^, all of which were of low quality, one concluded that there was inconclusive evidence for change in vegetable intake and two concluded that FV interventions had a positive effect on FV intake but these both included studies in primary/elementary school children. The two SR focusing on beverage intake^(27,28)^ both concluded that there was evidence for improvements in SSB and water intake; one SR was rated low quality and one was rated critically low. The high-quality SR by McHugh *et al.* concluded that there was limited evidence of improved dietary behaviours for nutrition interventions following the WHO Health Promoting Schools framework. Finally, the high-quality SR examining barriers and facilitators of dietary improvement^(30)^ concluded that there was mixed evidence for improvements in knowledge and dietary behaviours, with differences according to gender. Overall effectiveness as described by the SR is summarised in Table 2.

As adolescence is associated with a low intake of FV and high intake of SSB, evidence for effective interventions targeting these dietary behaviours, alongside knowledge of and attitudes towards nutrition, was synthesised.

#### Reported effectiveness in key dietary behaviours

##### Sugar-sweetened beverage/water intake

Two SR^(27,28)^ reported on the effects of interventions specifically to reduce SSB or increase water intake, and four SR included change in SSB consumption as one of a variety of dietary behaviours^(19,22,23,28)^. Overall, there is limited evidence that environmental restructuring involving reduction in the availability of SSB or increased availability of water may be beneficial. The SR by Vézina-Im *et al.* reported that 72 % of studies resulted in significant reductions in SSB, with legislative or environmental interventions being the most effective at prompting this change. However, authors describe that over 60 % of studies received a weak quality rating. The SR did not report on how SSB intake was measured, i.e. ml or servings per day. Calvert *et al.* reported eight studies that included SSB intake as an outcome and described that 75 % of studies resulted in significant improvements in dietary behaviours. However, in this SR, as well as the others that included SSB intake as one outcome amongst several dietary behaviours, it was not possible to identify intervention effectiveness specifically for SSB consumption.

##### Fruit and vegetable consumption

Three SR reported solely on FV interventions^(24–26)^ and eleven SR included FV consumption as a measurement outcome. Results from the SR with a focus on FV were mixed; however, provision of free FV was the most promising component. The SR by Nørnberg *et al.* reported on choice architectural nudge interventions involving the distribution of free vegetables and modifications to serving style and found limited effects on vegetable intake. The distribution of free vegetables was found to have a positive impact on attitudes towards vegetables and willingness to try them but no effect on vegetable consumption. Conversely, De Sa and Lock, who reported on school FV schemes, concluded that the provision of free or subsidised FV increased consumption, although this was not always sustained at follow-up. Knai *et al.* reported positive outcomes on FV intake in only one out of four studies on adolescents. The lack of success was suggested to be due to short intervention duration.

The remaining SR^(18–20,22,23,28–30)^ reported change in FV consumption as one of a variety of other dietary behaviours, including fat and sugar intake. Studies in these SR changed the school environment and increased the availability of FV through provision, and results suggested overall positive change in intake.

The majority of dietary interventions include FV intake as a single behaviour. However, Nørnberg *et al.* focused on interventions to increase only vegetable consumption in schools. This separation was deemed important given the higher intakes of fruit in adolescents compared with vegetables and the type of intervention that might be used to promote consumption.

##### Nutritional knowledge and attitudes

Seven SR^(20,21,23–26,30)^ reported on changes in nutrition knowledge and/or attitudes to food behaviours. Shepherd *et al.* concluded that 86 % of studies were effective at improving dietary behaviours and/or knowledge but increased knowledge was not consistent. Interventions involved classroom education, parental involvement, peer-taught lessons and changes to school meals, but authors did not identify specific intervention components that contributed to success. However, Shepherd *et al.* did identify gender- and age-specific results, reporting that interventions were more successful in females and 15- to 16-year-olds compared with 12- to 13-year-olds. The three studies that included nutritional knowledge as an outcome in the nutrition education-focused SR by Murimi *et al.* all reported improvements. De Sa and Lock reported one study in secondary schools with nutritional knowledge as a primary outcome, but no differences were found at follow-up. Robinson *et al.* reported four studies that included measurement of nutritional knowledge, all of which led to significant improvements in knowledge, but studies had multiple components and authors did not report on components contributing to improved nutritional knowledge.

As evidence is mixed for successful interventions specific to certain dietary behaviours, characteristics that contributed to successful interventions for multiple dietary behaviours were identified from the SR.

#### Intervention approaches identified in included systematic reviews as contributing to positive outcomes

SR identified a number of characteristics that contributed to the success of dietary interventions. Common components were increased availability of or exposure to healthy foods (six SR; ^(13,14,18–20,22)^); multicomponent interventions, i.e. education plus environmental restructuring (five SR;^(13,19–22)^); the use of online content/media and messaging (four SR;^(13,14,20,23)^); peer or parent involvement (four SR;^(13,14,20,22)^) and the use of behavioural theories (four SR;^(16,20,23,24)^).

##### Increased availability of or exposure to healthy foods

Six SR described improvements in outcomes following an increased availability of or exposure to healthy foods. One SR^(25)^ discussed that increasing availability of or exposure to healthy foods, specifically FV in this SR, could be achieved in a number of ways, including provision as snacks, a change in school meals, a school garden, healthy breakfast provision or via cooking or tasting sessions at school. FV intake was the most commonly explored dietary behaviour in relation to increased availability.

Linked to this are findings from three SR^(21,27,29)^ that approached the same issue from a different perspective, suggesting pursuing efforts to restrict access to unhealthier foods such as SSB and high energy or sugary snacks. This is based on evidence for reduced consumption following restriction of these foods in schools.

##### Multicomponent interventions

Interventions included in the SR can be characterised into three types: educational only (or behavioural), environmental only (or legislative/policy) and educational and environmental combined (multicomponent). The type of interventions included in each SR are summarised in Table 3.

**Table 3:** Summary of key variables

	Outcome measures (dietary)				Outcome measures (non-dietary)			Characteristics of interventions included in the review					
Reference	FV intake	SSB intake	Nutritional knowledge & attitudes	Other dietary behaviours e.g. fat intake	Anthropometry (BMI, body fat)	Process evaluation	Cost-effectiveness	Theory-based e.g. SCT	Include home-based intervention	Parental involvement	Peer involvement	Educational	Environmental
*High quality*													
Shepherd *et al.* (2006)	x		x	x	x	x		x		x	x	x	x
McHugh *et al.* (2020)	x			x	x	x	x	x		x		x	x
*Moderate quality*													
Van Cauwenberghe *et al.* (2010)	x			x	x	x		x		x	x	x	x
*Low quality*													
Knai *et al.* (2006)	x		x							x	x	x	x
De Sa *et al.* (2008)	x		x		x					x	x	x	x
Meiklejohn *et al.* (2016)	x	x		x	x			x		x	x	x	x
Nørnberg *et al.* (2016)	x		x					x					x
Vezina-Im *et al.* (2017)		x						x		x		x	x
Murimi *et al.* (2018)			x	x	x			x		x		x	x
Calvert *et al.* (2019)	x	x		x						x	x	x	x
*Critically low*													
Ajie *et al.* (2014)	x	x	x	x	x			x	x	x		x	
Robinson *et al.* (2014)	x		x	x	x					x	x	x	x
Vezina-Im *et al.* (2019)	x	x		x				x				x	x

FV, fruit and vegetable; SCT, social cognitive theory; SSB, sugar-sweetened beverage.

Five SR conclude that multicomponent interventions, i.e. those combining education and changes to the environment, are more successful than environmental or educational alone. However, Van Cauwenberghe *et al.* describe moderate evidence suggesting a benefit of educational-only interventions. Conversely, two SR by Vézina-Im *et al.* in 2017 and 2019 concluded that environmental or legislative interventions alone were more effective than educational or multicomponent interventions. In both SR, authors focused on a single dietary behaviour, i.e. SSB and water consumption, respectively. Therefore, although evidence is mixed and success may be dependent on the dietary behaviour targeted, multicomponent interventions were most commonly reported to be successful.

##### Use of online content/media and messaging

Four SR reported successful dietary improvements and increased knowledge following interventions incorporating media, computer-based feedback and messaging and/or online content. These components were deemed more ‘age appropriate’ by some SR and, therefore, relevant to a secondary school environment. The SR by Murimi *et al.* discussed internet use and multimedia CD, for example, as contributors to successful interventions. Ajie *et al.* reviewed computer-based interventions only, and recommended the use of tailored feedback. Similarly, almost all of the studies that employed personalised computer-based dietary feedback in the SR by Calvert *et al.* were successful in reducing intake of SSB and increasing FV, dairy product and protein intake. This personalised feedback involved comparison with standard behaviour towards foods such as dairy products. Additionally, seven studies in this SR included media content in the form of, for example, radio or television shows promoting healthy eating behaviours, and these all reported positive change in dietary behaviours such as FV intake.

### Peer or parent involvement

Four SR discussed peer and parent involvement as contributors to successful interventions. Interventions involving peers included peer-led education sessions, role models, group projects and discussions and consistent peer support. De Sa and Lock concluded that motivation from peers or fictional role models were features in three out of seven studies in adolescents that led to an increased intake of FV. The SR by Calvert *et al.* identified peer involvement as a main contributing factor to successful interventions, reporting that all nine studies that included peer involvement, the majority of which were rated moderate to high quality, were successful in promoting positive behavioural change. One SR (30) concluded that peers could effectively deliver nutrition education in schools.

Parents were involved in a number of ways across studies, including measurement of their own FV intake, health camps, invites to school meetings, homework assignments or by receiving written material. One SR^(21)^ suggested that face-to-face engagement with parents is necessary, as interventions that involved parents through, for example, nutrition classes or tasting sessions with pupils were more effective than passive methods such as receiving written material. However, SR and primary studies did not directly compare interventions with and without parental support.

### Theoretical basis of the intervention

Four SR reported the use of behavioural theories as a feature of successful interventions. The most frequently used theories were Social Cognitive Theory, Transtheoretical Model and the Theory of Planned Behaviour. However, the most effective of these upon which to base interventions could not be distinguished. SR often stated that one theory could not be described as more effective than others could, but overall it was recommended that the use of theory was better than no theory. One SR^(21)^, however, reported that the use of theories was not associated with success but discussed that this may be because the interventions were ‘informed by theory’ rather than ‘theory-driven’. This limitation is also discussed in one other SR^(15)^. Two SR^(27,28)^ attempted to code behaviour change techniques in interventions to identify those most relevant to effectiveness but neither were able to because of different combinations of behaviour change techniques used. However, the most frequently reported behaviour change techniques in the SR by Vézina-Im *et al.* (2017) were information about the health consequences of the behaviour, restructuring the physical environment, behavioural goal setting, self-monitoring of behaviour, threat to health and social support.

#### Other intervention characteristics

##### Population

None of the included SR reported if effectiveness was related to nationality or socio-economic status, as these were not examined in most SR. However, a number of SR reported gender differences in results^(19,23,24,30)^, concluding that many dietary interventions were solely effective in females^(23,24,30)^. In one SR^(19)^, it was reported that four studies targeted females only but none targeted males. Calvert *et al.* described that different genders responded to different intervention components, for example, girls increased fruit consumption while boys reduced snacking.

##### Intervention duration and exposure

Intervention duration ranged from a one-off session to three years. Three SR concluded that longer duration interventions were more successful at improving dietary behaviours, suggesting at least six months^(21)^ and 12 months^(24)^ duration and an average of 6·5 computer-based education sessions^(23)^. However, one SR^(20)^ concluded that duration did not impact study findings, although authors described that few interventions were longer than 12 weeks. One SR^(19)^ concluded that exposure to interventions was more important for effectiveness than duration, which is supported by conclusions drawn by Murimi *et al.* who further suggest that interventions with contact time in intervals longer than two weeks were less likely to be successful.

##### Intervention provider

SR did not provide evidence for comparison between specific intervention providers. However, there is evidence for effectiveness with various implementers, including teachers, researchers, nutrition professionals and peers, suggesting that a range of individuals can deliver successful interventions. One SR^(21)^ reported that providing training for the implementers contributed to the success of interventions, suggesting that the provision of resources was not enough to ensure fidelity and success.

### Limitations and recommendations

SR identified a number of limitations in the included primary studies, which have been summarised in Table 4. The most commonly reported were inconsistent measurement tools^(18–20,22,23,25–28)^, short duration or intensity of intervention^(18–21,23–25,28)^ and a lack of generalisability to other countries^(18,19,24–26,30)^. Table 4 also contains recommendations for future interventions as described by the SR. Recommendations from high- and moderate-quality studies are highlighted. The most common recommendations from SR were for consistency on measurement tools for dietary intake, addressing the environment outside as well as inside schools and taking demographics such as gender into account during interventions.

**Table 4:** Limitations and research recommendations

Limitations of primary studies included in the reviews	Inconsistent measurement tools^(18–20,22,23,25–28)^ *
	Short duration/low exposure^(18–21,23–25,28)^ *
	Not country generalisable^(18,19,24–26,30)^ *
	Use of self-report data^(18,20,22,24,25)^ *
	Lack of description/reporting^(19,21,27,29)^ *
	Low-quality ratings^(18,26,27,30)^ *
	Grouping of multiple behaviours^(19,21,25,27)^
	Lack of result or data at follow-up^(18–20,28)^ *
	No process or cost evaluation^(20,24,29)^ *
	No data outside schools^(18,25,27)^ *
	Lack of data in minority groups^(18,30)^ *
Recommendations from reviews for future interventions	Consensus about best dietary measurement tools^(18,19,22,26,27)^ *
	Address environment outside school to avoid unintended consequences^(18,19,22,27)^ *
	Tailoring interventions to take account of population demographics, e.g. gender^(18,19,26,30)^ *
	Greater length of or exposure to intervention^(18,19,21)^ *
	Explicit use of behavioural theory^(18,21,27)^ *
	Clear reporting of the BCT used for particular health behaviour^(19,21,27)^
	Multiple strategies to deliver same interventional messages^(19,22,29)^ *
	Introduce maintenance sessions and assessment at follow-up^(18,19)^ *
	Target intervention to specific behaviour rather than to broad promotion of healthy eating^(19,21)^
	More studies in adolescents^(18)^ *
	Face-to-face parental engagement^(21)^
	Train implementers to use resources^(21)^
	Examine attitudes to the interventions^(26)^
	Alignment of intervention with school’s core aims^(29)^ *

* Limitation or recommendation from high- or moderate-quality SR.

## Discussion

### Overall findings

This SR has systematically identified and synthesised evidence on components of successful interventions for improving dietary behaviours specific to secondary school environments. Results indicate that while interventions are heterogeneous, there are common characteristics that contribute to improved dietary behaviours in secondary school pupils. These are multicomponent interventions combining environmental and educational strategies, increased availability of healthy foods, multimedia and computer-based education and feedback, peer and/or parental involvement and the use of behavioural theory as a basis to the intervention. However, of the thirteen SR, only two received a high confidence rating and reported mixed evidence for overall improvements in dietary behaviours. It is important to highlight that this SR of SR aimed to review studies on food waste in the school environment, but no relevant studies were found for inclusion and few studies in this area overall were found.

### Targeting problematic dietary behaviours – fruit and vegetables and sugar-sweetened beverages

Although adolescents have been shown to have low intakes of FV and higher than recommended intakes of SSB^(34)^, the majority of SR included here report the results of interventions on multiple behaviours, making it difficult to discern how FV and SSB intakes, explicitly, are influenced. Vézina-Im *et al.* (2017) suggested that interventions aimed at multiple behaviours should clearly report which BCT was used for each behaviour, as the selection of BCT based on the behavioural theory behind the intervention was not always clear in the primary studies. Furthermore, finding interventions on established behavioural theories may improve effectiveness. Approaches that increase the availability of FV, for example, by free provision, may improve uptake, or at least attitudes towards FV, but it is likely that interventions such as this would need to be implemented long term. Cost-effectiveness and adherence would then need to be assessed. Considering fruit and vegetable intakes as separate dietary behaviours, which was suggested by two SR and supported by other literature^(35)^, is likely to have an impact on how environmental interventions that involve restructuring of the physical environment or provision of FV are implemented. This may be an important consideration for future interventions.

Similarly, environmental changes including an increase or decrease in availability of a single dietary component, i.e. SSB or water intake were reported by two SR to be more effective than education in influencing intake. However, it was cautioned that implementing only environmental change may increase the risk of unintended consequences associated with the intervention, described by Von Philipsborn *et al.* as adverse compensatory behaviour^(36)^. Interventions that restrict certain foods or beverages at school may lead to increased consumption before and after school. Nutrition education alongside environmental restructuring may mitigate the risk of these unintended consequences. A larger number of SR included here concluded that multicomponent interventions combining environmental changes with education were more successful. Furthermore, a review on SSB interventions by Avery *et al.* ^(37)^, which was not included in this SR, highlights nutrition education delivered by peer, teachers or nutritionists can be effective at reducing SSB consumption. However, in general positive results were not maintained at follow-up. This may be due to short-term effects of education-only interventions and highlights a need for maintenance sessions as well as environmental restructuring alongside education.

### Targeting adolescents

The development from child to adolescent is associated with more independence, fuller schedules, eating away from home, growing concern over appearance and weight and peer acceptance^(12)^, all of which influence dietary behaviours^(38)^. Several SR and a SR of SR on physical activity in children and adolescents^(39)^ highlight the small number of studies conducted in secondary schools compared with primary schools, weakening the evidence from SR on adolescents. For example, Murimi *et al.* reported that only 20 % of their studies took place in secondary schools. Addressing this imbalance with more interventions targeting secondary school settings will strengthen the evidence base for interventions that positively influence the dietary behaviours of a range of age groups. Considering interventions in secondary schools separate to those in primary schools will also allow the development of age-appropriate strategies, such as those that require more developed cognitive skills like receiving personalised dietary feedback. Furthermore, results from this SR have highlighted that peers can successfully lead interventions, strengthened by process evaluations included in the high-quality SR by Shepherd *et al.* that suggest that interventions are acceptable to both the peer leaders and the receivers. This reiterates the process evaluation results of a peer-led education study, which concluded that peer-led nutrition education is feasible and well accepted by pupils and teachers^(12)^. This educational strategy may be a useful tool in the secondary school environment.

Gender differences in body image and body composition also become more apparent in adolescence^(40)^. Khambalia *et al.* ^(14)^ discuss that male and female motivations and responses to intervention components differ, which was apparent in results from three SR included here^(19,23,30)^, as well as a SR not included^(41)^. Significant effects were often seen in females only, suggesting that gender-specific intervention components should be further explored.

### Limitations and research gaps

The majority of primary studies lacked process or cost-effectiveness evaluations. This echoes the findings of other SR of SR on the control and prevention of obesity and interventions to reduce free sugar intake^(14,42)^. A lack of information on acceptability and feasibility for in-school interventions limits their ability to be effectively implemented. Although it has been suggested that interventions delivered by non-school individuals are too expensive and unsustainable^(43)^, McHugh *et al.* caution unnecessarily burdening school staff and curricula with interventions that do not align with the school’s priorities. These together suggest that there is a balance to strike between the cost of non-school staff as implementers of the intervention, and time and resource constraints of school staff if expected to deliver the intervention themselves. In addition, retention of knowledge and maintenance of dietary patterns in the secondary school environment is currently unknown, as interventions tended to lack long-term follow-up. Future research should consider maintenance sessions and adequate follow-up when designing interventions.

Some other limitations of the primary studies included heterogeneity in measurement tools, self-report data and a lack of description or selective reporting. However, as described by Meiklejohn *et al.*, self-report data collection may be the only time- and cost-effective method for use in school-based interventions. An additional criticism of individual studies was that implementation, fidelity and participation rates were frequently not reported. This limits the development of recommendations for future interventions and the ability of decision makers to stimulate changes in practice. Reporting of participation and fidelity may help to explain why some interventions were ineffective at promoting healthful dietary behaviours. Furthermore, reviewed interventions were mostly conducted in Europe and the USA, and, therefore, may not be generalisable. This is particularly important considering that dietary guidelines differ between countries and, thus, studies in less developed countries are needed.

Finally, this SR aimed to synthesise evidence on reducing food wastage in the secondary school environment, but no relevant SR were found in this area. One review^(44)^ discussed inconsistent measurement techniques for food wastage in the National School Lunch Program in the USA and similar to SR included in this SR, report on few studies in post-primary/elementary school. A review by Reynolds *et al.* reported on three positive school-based interventions on food waste reduction involving changing dietary guidelines and education on food waste^(45)^, but these were either in primary school or the age group was not reported. In conclusion, analysis of food wastage in secondary school food environments is lacking and future research should include an aspect of food waste analysis, given the negative impact it can have on nutrient intake in pupils^(46,47)^.

### Quality of the systematic reviews

The majority of included SR received a low or critically low-quality rating. However, it must be noted that some SR were conducted before the quality assessment tool, AMSTAR, was published and updated in 2007 and 2017, and so less guidance was available for conducting SR at the time. Furthermore, the domains of AMSTAR-2 are open to interpretation and, thus, ratings do not represent a universal quality score. Based on the research setting of the present SR, not all critical domains advised by Shea *et al.* ^(16)^ were applied. Despite this, many SR had fundamental flaws such as not accounting for the quality of primary studies when interpreting results. One critical domain applied in assessment was that there was explanation of study designs included in the SR. For public health interventions such as those targeting dietary behaviours, randomised controlled trials may not always be achievable and appropriate^(48,49)^, particularly in a school setting, and do not necessarily increase the quality of evidence^(50)^, so it is important that authors explain their inclusion of one or multiple study designs in SR.

### Strengths and weaknesses

Results from this SR have generated a comprehensive overview of intervention components specific to secondary school environments that have been reported to contribute to successful interventions. Nevertheless, this SR is not without limitations. The SR included in this SR were generally of low quality, lacking explanation of included study designs and not taking risk of bias into account when synthesising evidence from primary studies. Therefore, results should be interpreted with caution. Furthermore, although the aim was to investigate interventions in secondary schools only, a number of SR included interventions in primary schools and, thus, some of the conclusions drawn about intervention effectiveness in secondary schools may overlap with those that apply more generally to all educational levels or to primary schools. However, due to strict inclusion criteria, this risk was minimised. There may also be sample bias driven by the higher numbers of studies, and therefore SR, in primary schools than secondary schools.

## Conclusion

There is currently limited evidence on school-based dietary interventions that can positively influence the dietary behaviours of adolescents. No single intervention type appears more effective than others in this setting and age group. Interventions should consider a design that incorporates a number of key characteristics that have been repeatedly reported to improve chance of success. These include the combination of education and environmental change, a theoretical basis, the use of ‘age-appropriate’ formats such as computer-based feedback, media and messaging, an increase in the availability of healthy foods and the involvement of peers and/or parents in education or support roles. Future research studies and interventions based on them would benefit from process evaluations and cost-effectiveness analyses for a variety of intervention durations and intensities, as well as implementers. This would guide the development of interventions on dietary interventions that have a positive effect on food choices in secondary school pupils without placing excessive burden on an already demanding school system.

## Supplementary material

For supplementary material accompanying this paper visit https://doi.org/10.1017/S1368980022000829. click here to view supplementary material
