# Supplementary material for: What makes interventions aimed at improving dietary behaviours successful in the secondary school environment? A systematic review of systematic reviews
Source: Public Health Nutr. 2022 Mar 31;25(9):2448–64. doi: 10.1017/S1368980022000829 (PMC9991643; doi:10.1017/S1368980022000829)
Supplement: Supplementary file 1 [file S1368980022000829sup.zip › S1368980022000829sup001.docx]

**Additional File 1: search terms and strategy**

The literature search was performed in May 2018 and updated in May 2020. The search terms are provided below in the updated search strategy for PsychINFO.

1. (food* or meal* or lunch* or snack* or eating or breakfast* or beverage* or breakfast club* or canteen* or cafeteria* or recess or after school or environ* or ecol* or green behavio?r or sustain* or local food* or local produce or vegetab* or fruit* or farm food* or local* grown or allotment or health promot* or health polic* or nutrition* educat* or nutrition* promo* or nutrition* polic* or diet* educat* or diet* promot* or diet* polic* or cook* educat* or cook* demo* or food prep* or school* health* or health* curric* or diet* curric* or food* waste* or household* waste* or plate* waste* or domestic waste* or leftover* or food* leftover or food choice or food preference or food selection or health choice or health preference or health selection or meal choice or meal preference or meal selection or snack choice or snack preference or snack selection, nutrit* habit* or nutrit* behavio?r* or nutrit* lifestyle* or nutrit* quality or nutrit* pattern or diet* habit* or diet behavio?r or diet* lifestyle* or diet*).mp. [mp=title, abstract, heading word, table of contents, key concepts, original title, tests & measures]
2. limit 1 to (human and English language and adolescence <13 to 17> and reviews and yr="2018 -2020")

For MEDLINE, search was limited to (English language and humans and year="2000-current" and "adolescent (13 to 18 years)" and systematic reviews).

For EMBASE, search was limited to (human and English language and year="2000-current" and "review" and adolescent <13 to 17 years>).

For CINAHL, search was limited to Publication Year: 2000-2018; English Language; Publication Type: Systematic Review; Human.

For Cochrane Database of Systematic Reviews, search was limited to systematic reviews and studies published between 2000 and 2018 only, as other limits were not available.

For Web of Science, search was limited to reviews, articles published between 2000-2018 and research areas: ‘Pediatrics’, ‘Public Environmental’, ‘Occupational Health’, ‘Nutrition Dietetics’, ‘Environmental Sciences Ecology’ or ‘Food Science Technology’ or ‘Behavioural Sciences’, ’Health Policy Services’, ‘Environmental Studies’, ‘Green Sustainable Science Technology’.
